# Autophagy in Spinocerebellar Ataxia Type 3: From Pathogenesis to Therapeutics

**DOI:** 10.3390/ijms24087405

**Published:** 2023-04-17

**Authors:** Rodrigo Paulino, Clévio Nóbrega

**Affiliations:** 1 ABC-RI, Algarve Biomedical Center Research Institute, Universidade do Algarve, 8005-139 Faro, Portugal; 2 FMCB, Faculdade de Medicina e Ciências Biomédicas, Universidade do Algarve, 8005-139 Faro, Portugal

**Keywords:** Machado–Joseph disease, spinocerebellar ataxia type 3, autophagy, ataxin-3, neurodegeneration

## Abstract

Machado–Joseph disease (MJD) or spinocerebellar ataxia 3 (SCA3) is a rare, inherited, monogenic, neurodegenerative disease, and the most common SCA worldwide. MJD/SCA3 causative mutation is an abnormal expansion of the triplet CAG at exon 10 within the *ATXN3* gene. The gene encodes for ataxin-3, which is a deubiquitinating protein that is also involved in transcriptional regulation. In normal conditions, the ataxin-3 protein polyglutamine stretch has between 13 and 49 glutamines. However, in MJD/SCA3 patients, the size of the stretch increases from 55 to 87, contributing to abnormal protein conformation, insolubility, and aggregation. The formation of aggregates, which is a hallmark of MJD/SCA3, compromises different cell pathways, leading to an impairment of cell clearance mechanisms, such as autophagy. MJD/SCA3 patients display several signals and symptoms in which the most prominent is ataxia. Neuropathologically, the regions most affected are the cerebellum and the pons. Currently, there are no disease-modifying therapies, and patients rely only on supportive and symptomatic treatments. Due to these facts, there is a huge research effort to develop therapeutic strategies for this incurable disease. This review aims to bring together current state-of-the-art strategies regarding the autophagy pathway in MJD/SCA3, focusing on evidence for its impairment in the disease context and, importantly, its targeting for the development of pharmacological and gene-based therapies.

## 1. Introduction

Machado–Joseph disease (MJD) or spinocerebellar ataxia 3 (SCA3) is a rare, inherited neurodegenerative disease belonging to a group of nine diseases known as polyglutamine (PolyQ) diseases. This group of diseases originates from an abnormal expansion of the triplet CAG (cytosine–adenine–guanine) in the coding region of different and unrelated genes. The expansion translates into a stretch of glutamine in the encoded proteins, which in the case of MJD/SCA3 is ataxin-3.

PolyQ diseases also include SCA1, SCA6, SCA7, SCA17, Huntington’s disease (HD), dentatorubral pallidoluysian atrophy (DRPLA), and spinal and bulbar muscular atrophy (SBMA). The diseases are characterized by progressive neurodegeneration in brain regions such as the spinal cord, resulting in motor, cognitive, and psychiatric signs and symptoms. MJD/SCA3 is the second most prevalent disease in the PolyQ group, affecting about 1–5 per 100,000 people globally [1]. However, in certain regions, such as the Portuguese archipelago of Azores, the prevalence is much higher [2].

The ataxin-3 protein is encoded by the *ATXN3* gene, which has a polyglutamine stretch that varies from 13 to 49 repetitions in its normal form [3]. However, in MJD/SCA3 patients, the size of the polyglutamine stretch increases from 55 to 87, leading the protein to adopt an abnormal conformation and promoting its aggregation and cellular toxicity, which eventually results in neuronal damage and loss [4]. Studies describe that the size of polyglutamine expansion positively correlates with the severity of the signs and symptoms, while it inversely correlates with the age of the onset of symptoms [3,5]. Clinical features such as pseudoexophthalmos and pyramidal signs are more frequent in the presence of higher expansion, indicating a complete penetrance [5]. However, due to MJD/SCA3 incomplete penetrance, the probability of an individual carrying the mutation without expressing any symptom decrease during their life diminishes to almost 0 at 70 years old [5].

MJD/SCA3 is a multisystem neurodegenerative disorder that affects several systems and regions, predominantly the cerebellum and pons, but also the *substantia nigra* and the striatum [5]. These regions’ impairment results in external symptoms, such as ataxia and parkinsonism, progressive ophthalmoplegia (EPO), dystonia, intention fasciculation-like movements of facial and lingual muscles, as well as bulging eyes [5,6]. Currently, there are no treatments able to modify the disease progression, which culminates in the patient’s death. Therefore, there is an urgent and unmet need for the development of therapies that could stop or delay the disease’s progression. Relatedly, the activation of autophagy emerges as a preferential molecular target for therapy, both based on pharmacological approaches and in gene-based delivery. This review focuses on research efforts made in the past few years to investigate different strategies to activate autophagy as a therapy in different MJD/SCA3 models.

## 2. The *ATXN3* Gene and Ataxin-3 Protein

The *ATXN3* gene, located on chromosome 14q32.1 and under non-pathological circumstances, has a triplet expansion of CAGs on exon 10 [3]. The gene encodes for the ataxin-3 protein, which carries the glutamine expansion in its C-terminal region.

**Figure 1:**
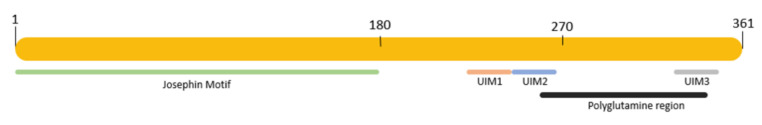
Representation of ataxin-3 protein with its main domains. Green, Josephin motif, positions 1–180; orange, UIM1 motif, positions 224–243; blue, UIM2 motif, positions 244–263; gray, UIM3 motif, positions 331–349; black, polyglutamine region, positions 258–338.

Ataxin-3 protein (Uniprot #P54252) has a molecular weight of approximately 42 kDa and four main domains, including the Josephin domain and three ubiquitin interacting motifs (Figure 1). The Josephin domain is located at the N-terminal of the protein between positions 1 and 180 and is believed to be the catalytic domain of the protein as it is widely conserved among species [7,8]. The three ubiquitin interacting motifs are located at the C-terminal region, are responsible for recognizing and interacting with ubiquitin, and have a higher affinity for ubiquitin chains composed of three or more residues [7]. The polyglutamine tract of ataxin-3 is located from positions 258 to 338 in its normal conformation. Ataxin-3 protein has at least two different splicing forms, both of which contain the polyglutamine domain and have been found in MJD/SCA3 patients [7].

Ataxin-3, under normal conditions, is a cytoplasmatic protein [9]. However, in MJD/SCA3 patients, it is also found in the nucleus, forming aggregates which are a neuropathological hallmark of the disease [10]. This protein uses specific translocators to move in and out of the nucleus, which is a feature that is mediated by nuclear export signals [11]. The reason why the protein is found inside the nucleus in the disease context is still unknown; however, this feature is not exclusive to MJD/SCA3 patients as it is also reported in other PolyQ diseases, such as HD [12].

Ataxin-3 is a deubiquitinating enzyme that acts on proteins that have been targeted for degradation through the ubiquitin–proteasome system, which cleaves the ubiquitin present on those proteins moments before degradation and allows the recycling of ubiquitin [13]. Additionally, the role of ataxin-3 in cytoskeleton regulation due to its interaction with tubulin and the microtubule-associated protein 2 has been described. Along with this, it is also suggested that ataxin-3 plays a role in transcriptional regulation due to its interaction with proteins related to aggresomes, such as dynein and histone deacetylase 6 (HDAC6) [4]. Additionally, the interaction of ataxin-3 with transcription factors and co-regulators has been described. It was shown that it interacts with the forkhead box O (FOXO)-4 transcriptional factor (FOX04), which is a member of the forkhead family of transcriptional factors that are related to gene expression regulation [14]. It also interacts with CBP, which is a co-activator that originates a nuclear response by cascade signaling [15]. It interacts with P300 [16], which is a histone acetyltransferase recruited for the enhancement and regulation of gene expression [17]. It was also shown that it interacts with histone deacetylases (HDAC3), which is a class I HDAC that is recruited, and deacetylase in the gene promoter region, leading to chromatin compaction and the formation of a transcriptionally repressive state preventing the transcriptional machinery from accessing the promoter region [18]. It interacts with the nuclear co-repressor receptor NCor, which contains several nuclear receptor interacting domains that play a role in the recruitment of histone deacetylases to DNA promoter regions that assist with gene down regulation [19]. In addition, ataxin-3 interacts with RAD32, which is a nucleotide excision repair protein that inhibits the degradation of a proteolytic substrate and multi-ubiquitin chain formation [20] (Table 1). These and other interactions suggest that ataxin-3 is involved in the regulation of a wide range of genes, including those involved in development, metabolism, and disease [10].

### Ataxin-3 Pathological Aggregation

PolyQ diseases are characterized by the formation of protein aggregates in neurons, which is a hallmark of the disease [21]. These aggregates form due to the adoption of a non-native conformation that becomes toxic for the cells or induces toxicity by nonspecific interactions [22]. Pathological aggregates can include other proteins and cellular components, such as transcription factors, molecular chaperones, and components of cellular clearance mechanisms [21]. Progressively, these aggregates become insoluble, ultimately contributing to the dysregulation and impairment of several cellular mechanisms, including the dysregulation of calcium homeostasis, ubiquitin–proteasome dysfunction, mitochondrial dysfunction, axonal impairment, aberrant protein interaction, proteolytic cleavage, post-translational modification alterations, autophagy impairment, and others that contribute to neuronal degeneration and death [23].

## 3. Autophagy

**Figure 2:**
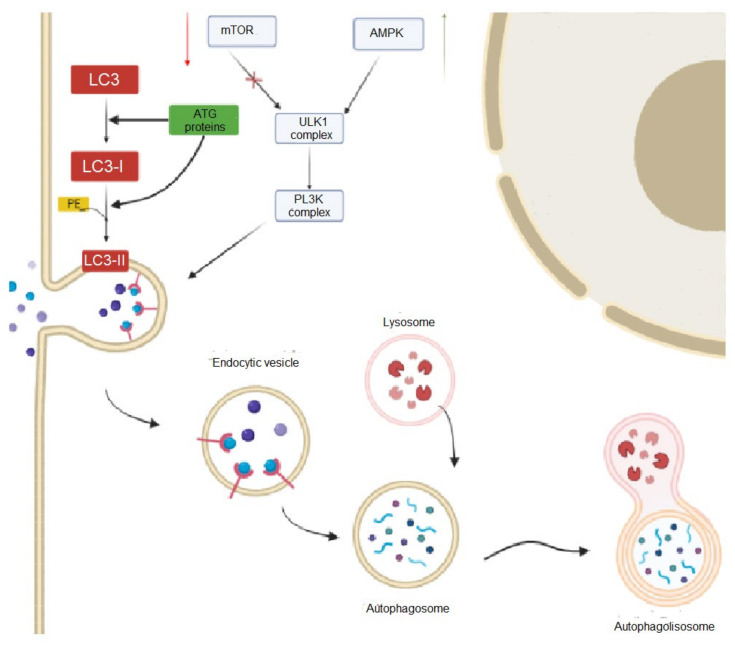
Overview of the main steps of macro-autophagy in mammal cells. In this pathway, the process of lysosome delivery is assisted by a double membrane-bound vesicle and the autophagosome that fuses with it to form the autolysosome.

Autophagy is a process by which cells maintain homeostasis by degrading and recycling cellular components in response to the deprivation of nutrients or oxygen or general damage [24]. Currently, three different types of autophagy are described that share a similar role but have differences in their pathways. Despite being different pathways, they all act to promote the proteolytic degradation of cytosolic components at the lysosome. In macro-autophagy, commonly named autophagy, the process of lysosome delivery is assisted by a double membrane-bound vesicle and the autophagosome that fuses with it to form the autolysosome (Figure 2). The autophagosome is made of a portion of the cellular membrane; thus, macro-autophagy is a non-selective degradation system, and this is a major difference when compared with other systems such as the ubiquitin–proteasome system (UPS) which act exclusively on proteins that have been ubiquitinated [25]. However, in micro-autophagy, cellular structures are directly taken up by the lysosome through the invagination of the lysosomal membrane. Finally, chaperone-mediated autophagy (CMA) uses chaperones to select proteins individually through a recognition motif in their amino acid sequences, translocating them to the lysosome for degradation [26].

Autophagy’s initial steps are crucial for the correct development of this degradation pathway. In these initial steps, the Unc-51-like kinase 1 (ULK1) complex plays an important role. The complex is formed by ULK1, ATG13, FIP200, and ATG101 proteins which are regulated by the mTOR complex 1 (mTORC1) signaling mechanism. mTORC1 inhibits autophagy according to the availability of nutrients in the cells, namely under nutrient-rich conditions. On the contrary, in depleted nutrient conditions, mTORC1 is inhibited and ULK1 is activated, initiating the autophagy process [24]. After, the autophagy elongation step involves the recruitment of ATG proteins to the phagophore. The formation of the phagophore comprises the formation of ubiquitin-like protein conjugated systems, including ATG12, ATG5, ATL161L1, the Ublike LC3 family, and the phosphatidylethanolamine (PE) which are present on the phagophore membrane. The elongation phase ends with the closure of the phagophore membrane, resulting in the formation of the autophagosome [24]. During the maturation phase, the autophagosome fuses with the lysosome, which is a process regulated by rab7 and LAMP-2, creating the autolysosome. Defects in the autophagy process have been linked to a range of diseases, including cancer, neurodegenerative diseases, and metabolic disorders.

### 3.1. Autophagy in MJD/SCA3

Autophagy is the main pathway responsible for removing oligomers or fibrils in the brain of neurodegenerative diseases patients as the UPS system is not able to degrade such large protein aggregates. However, the impairment of autophagy is a common feature in neurodegenerative diseases which compromises this degradation. Mutations in genes related to the autophagy pathway are linked to the development of familial neurodegenerative diseases. Moreover, in idiopathic neurodegenerative diseases, the presence of protein aggregates hinders autophagic degradation even without mutations [27]. In polyglutamine diseases, there is much evidence of an impairment of autophagy, which plays an important role in disease pathogenesis [28,29].

As described, autophagy is a highly regulated pathway in which several proteins are involved. For example, beclin-1 is important for the localization of autophagic proteins to a pre-autophagosomal structure [30]. Ataxin-3 protein was shown to protect beclin-1 from proteasomal degradation by interacting with it through the ataxin-3 polyQ stretch, thus enabling autophagy [31]. This study also showed that cells depleted of ataxin-3 showed partial inhibition of autophagy. Moreover, results suggest that a lack of or malfunction of ataxin-3 causes decreased autophagy as no interaction exists with beclin-1 [31].

Previously, it was shown that several autophagic proteins abnormally accumulate in the brain of MJD/SCA3 patients [32], including autophagic proteins LC3-II and p62/SQSTM1 [33]. Later, Onofre and colleagues used fibroblasts derived from MJD/SCA3 patients and reported that beclin-1 mRNA and protein levels were reduced in patients compared with healthy controls [34]. LC3 is a protein that is present on the lumen and cytosolic surface of mature autophagosomes. It normally exists as LC3-I form until it is conjugated with phosphatidylethanolamine (PE), forming LC3-II. This latter form is recruited to the membrane of the autophagosome to assist the process of fusion between the autophagosome and lysosome [35]. However, there is no consensus about the expression levels of LC3-II in MJD/SCA3 as some findings found increased levels, while other studies showed reduced LC3-II expression levels [34]. p62/SQSTM1 is involved in several signal transduction pathways shuttling cargo for autophagic degradation. It was shown to have an abnormal accumulation in MJD/SCA3 patients’ fibroblasts [34]. It is an autophagy substrate which acts as an adapter protein that binds to ubiquitinated protein aggregates and targets them for autophagic degradation. Impairments in autophagy could be related to increased levels of p62/SQSTM1, which resulted in an increase of polyubiquitinated aggregates in Drosophila and mammal models [36].

### 3.2. Targeting Autophagy through Pharmacological Approaches

Several pre-clinical studies and clinical trials were conducted in the last decades to try to find pharmacological therapies for MJD/SCA3 by targeting the activation of autophagy (Table 2). However, generally, the results obtained show a mild improvement in motor deficits and neuropathological abnormalities (Table 3). In 2002, the efficacy of rapamycin was investigated as an inducer of cell clearance mechanisms, especially autophagy, targeting the degradation of aggregate-prone polyglutamine proteins. Using a cellular model, the efficacy of rapamycin treatment was evaluated at 24 and 48 h after transfection with plasmids that were encoded for an expanded polyQ protein. The 24 h treatment with rapamycin led to a decrease in cell death and in the proportion of cells containing aggregates. However, in the 48 h treatment, no significant improvement was observed upon rapamycin treatment. These results suggest that in advanced stages, the consolidated structure of aggregates adopt a stable form that confers resistance to autophagy induced by rapamycin [37,38]. In the same study, researchers used an adrenal phaeochromocytoma cell line (PC12) expressing EGFP-Q74 to evaluate the efficacy of rapamycin in reducing protein levels and aggregates. The rate of protein clearance improved when it was treated with rapamycin and showed a reduction in GFP-positive fluorescence. Through western blot, the study showed a reduction both in the soluble and aggregated protein with rapamycin treatment [37]. It was hypothesized that rapamycin could be acting as a slow inhibitor of protein synthesis. However, when using cycloheximide, which is a protein synthesis inhibitor, it was possible to obtain an increase in protein levels and a decrease in autophagy activity [36].

Based on previous findings, a study used temsirolimus, which is a rapamycin ester that is soluble in water, inhibits mTORC, and increases and upregulates protein degradation by autophagy. The study used primary cortical cells and MJD/SCA3 transgenic mice at 5 weeks of age [40]. As temsirolimus acts as a kinase mTOR inhibitor, researchers investigated if the mTOR pathway was in fact inhibited in the brains of treated mice. Under normal conditions, LC3-II levels could be used to evaluate whether autophagy was being induced as LC3-II is a key component in the formation of autophagosomes. However, LC3-II levels in vivo were impossible to access, probably because neurons clear the autophagosomes. Therefore, researchers focused on the expression of two different mTOR pathway substrates, including p7056 kinase and eukaryotic initiation factor 4E-binding-protein-1, which were assessed by western blot. The results showed a decreased expression in the temsirolimus-treated group compared with the control animals. Additionally, an improvement in treated mice performance was observed with a higher latency to fall in the rotarod compared with the controls. The neuropathological analyses showed a reduced number of aggregates in the motor cortex and a reduction in the cytoplasmatic levels of expanded ataxin-3 in the treated animals. However, the levels of nuclear inclusions remained the same in the treated and control groups, probably because autophagy is a cytoplasmic process [42].

The activation of the monophosphate-activated protein kinase (AMPK) pathway is one of several pathways able to induce autophagy. A study used cordycepin, which is an AMPK inducer, to active autophagy in cellular and mouse models of MJD/SCA3. Researchers reported that N2a cells treated with cordycepin presented increased levels of phosphorylated-AMPK (P-AMPK) and a decrease in general protein synthesis. As the AMPK pathway can induce autophagy by itself, the authors evaluated whether cordycepin activated the AMPK pathway and autophagy. In neuroblastoma cells (N2a) expressing mutant ataxin-3, cordycepin treatment (20 µM) reduced the levels of the protein, while it did not interfere with the levels of non-expanded ataxin-3 (both wild-type ataxin-3 human and mouse endogenous ataxin-3). In addition, the levels of LC3B-II and p62/SQSTM1 reduced in the treated cells, indicating an increase in autophagy clearance. Treatment with cordycepin was also evaluated in induced pluripotent stem cells (iPSC) derived from MJD/SCA3 patients. The results were in line with those reported for the N2a cells, showing fewer aggregates and higher cell viability upon cordycepin treatment. Moreover, in an MJD/SCA3 lentiviral mouse model [41], cordycepin administration (20 mg/Kg) was able to reduce the levels of insoluble and soluble expanded ataxin-3. In addition to that, a decrease in the total levels of ubiquitinated aggregates and their size was observed due to an increase in the autophagy flux upon cordycepin treatment. These results were accompanied by preservation in the neuronal marker NeuN, showing signs of neuroprotection [39]. Finally, in a transgenic mouse model [43], cordycepin treatment for six weeks (starting from 15 mg/Kg to 25 mg/Kg) showed a higher number of Purkinje cells and a reduction in the number of aggregates compared with the control animals that were treated with saline solution. The results also showed an increased latency to fall on the rotarod and improvements in the footprint patterns of the cordycepin-treated animals [39].

In 2020, another study evaluated the trehalose efficacy as an autophagy inducer in the treatment of MJD/SCA3. In this study, 10 mM of trehalose was applied to N2a cells expressing mutant ataxin-3 for different periods of time. The levels of LC3B as an autophagy marker were measured, along with the levels of the human mutant ataxin-3. Western blot analyses showed an increase in the expression of LC3B-I and LC3B-II in the conditions treated with trehalose, indicating the activation of autophagy. The results also showed a decrease in the levels of mutant ataxin-3 at 24 h, 48 h, and 72 h post-treatment with trehalose. In vivo, the study reported the rescue of motor and coordination of an MJD/SCA3 transgenic mouse model [43] treated with a 2% trehalose solution for 28 weeks. The trehalose-treated animals showed an improvement in their rotarod performance and a decrease in their ataxia phenotype compared with the control animals. These results are in line with neuropathological findings as trehalose-treated animals showed the preservation of Purkinje cells and a decrease in the number and size of mutant ataxin-3 aggregates. However, is worth mentioning that the behavior tests and neuropathological analyses were performed just in female mice which are characterized by a weaker phenotype [44].

Carbamazepine (CBZ), which is a medicine used for the treatment of epilepsy, nerve pain, and bipolar disorder, was investigated as a therapeutic option for MJD/SCA3 through autophagy activation. Vasconcelos-Ferreira and colleagues showed that carbamazepine acts through an mTOR-independent pathway, leading to AMPK activation. In this study, three different strategies were tested considering the length of the treatment and the administration frequency. CBZ treatment was administered to MJD/SCA3 transgenic mice for a short period of time (1 week), a long period of time (4 weeks), and intermittently (three times per week) for 10 weeks [45]. The authors found that the animals that were treated with carbamazepine for a continuous long treatment (50 mg/Kg) did not present significant improvements in neuropathology or behavior deficits. Moreover, the results showed that both p62 and AMPK phosphorylation levels were not changed, suggesting that autophagy was not induced. However, the short and intermittent administrations were more efficient. In vitro, cells treated with CBZ (100 µM) showed a significant reduction in the levels of high molecular weight species of mutant ataxin-3. In vivo, the short treatment led to a reduction in the high molecular weight species of mutant ataxin-3 both in the striatum and in the cerebellum. In the intermittent treatment, animals that were treated with 50 mg/Kg of CBZ showed significant improvements in their rotarod performance from 4 weeks to 8 weeks of treatment. This was accompanied by a significant improvement in footprint overlapping at 4 weeks of treatment, suggesting a rescue of balance and coordination. Along with this, a decrease in the number of aggregates, a higher number of Purkinje cells, and the preservation of molecular layer thickness were observed in the CBZ-intermittently treated mice compared with the control animals [45]. 

The small molecule n-Butylidenephthalide (n-BP) isolated from the plant *Angelica sinensis* was investigated as a therapeutic approach for MJD/SCA3 using a transgenic mouse model [46]. In this study, the animals that started presenting motor deficiency at 13–14 weeks of age were treated with n-BP at 23 weeks of age until the animals reached 28 weeks of age. The treated and control animals were subjected to behavior tests to assess their balance and coordination through accelerated rotarod and footprint patterns tests. The animals treated with n-BP presented a significant improvement in their distance traveled, latency to fall, and speed when compared with the non-treated group, yet they never reached the performance of wild-type mice. The footprint patterns results reinforced these results as n-BP treated mice presented a closer relationship with a normal phenotype, showing a shorter footprint overlap and a longer stride length compared with the control animals [47]. The highest improvement observed in all the tests was in the fourth and fifth weeks of treatment, indicating that longer exposure to treatment is beneficial. The neuropathological analysis showed that the treated animals had a larger brain volume compared with the non-treated group and preserved the number of Purkinje cells. Moreover, the neurite network in the cerebellum was preserved, showing non-fragmented signs of neurite in the animals treated with n-BP, which is similar to what is observed in wild-type animals [47].

### 3.3. Targeting Autophagy through Gene-Based Approaches

As described above, several studies were performed using pharmacological strategies to activate activation autophagy. Similarly, recent gene-based approaches were also used in a preclinical setting to activate autophagy as a therapeutic option for MJD/SCA3 (Table 4). Overall, gene delivery strategies to activate autophagy improve neuropathological abnormalities and behavior deficits in MJD/SCA3 mouse models (Table 5). In 2011, a study analyzed levels of the autophagy protein beclin-1 in MJD/SCA3 patients’ fibroblasts. The results showed a decrease in beclin-1 levels compared with fibroblasts from healthy controls. Based on that, the authors prompted to investigate whether the up-regulation of beclin-1 could lead to an improvement in the MJD/SCA3 disease phenotype [32]. In primary striatal cells, beclin-1 expression led to a drastic reduction of mutant ataxin-3 aggregates, oligomers, and soluble protein. Additionally, in an MJD/SCA3 lentiviral mouse model [41], beclin-1 expression mediated by lentiviral vectors showed a reduction in the number of aggregates at 4 weeks and 8 weeks post-injection. Similar findings were observed in the neuronal dysfunction upon beclin-1 up-regulation. Altogether, these results suggest that the overexpression of beclin-1 mitigates MJD/SCA3 neuropathology [32].

Another study using beclin-1 was performed using an MJD/SCA3 transgenic mouse model with a severe motor phenotype [43] and aimed to evaluate the efficiency of the gene-based delivery of beclin-1 to induce autophagy and revert the disease phenotype. In this study, beclin-1 expression was mediated by lentiviral vectors which were injected in 4 week old animals. At 10 weeks post-injection, the beclin-1 injected animals showed an improvement in motor behavior, namely in the rotarod and beam walk test compared with the control animals injected with GFP [48].

In MJD/SCA3 patients, several molecular mechanisms are affected in neurons and underlie disease pathogenesis. In 2019, a study was published focusing on cholesterol 24-hydroxylase (CYP46A1). This is an enzyme that is responsible for converting cholesterol into 24S-hydroxycholesterol, allowing the efflux of brain cholesterol, and activating brain cholesterol turnover. When CYP46A1 levels are reduced, it can lead to a malfunction causing the accumulation of cholesterol in the brain. The reduction of this enzyme seems to occur in several neurodegenerative diseases, including MJD/SCA3 [49]. In this study, Nóbrega and colleagues evaluated whether restoring CYP46A1 expression could lead to improvement in the MJD/SCA3 disease phenotype. In an MJD/SCA3 lentiviral mouse model [41], the overexpression of CYP46A1 promoted the clearance of mutant ataxin-3 aggregates and neuroprotection [49]. Similarly, in a transgenic MJD/SCA3 mouse model [43], the upregulation of CYP46A1 in the cerebellum led to an alleviation of the motor phenotype and neuropathological abnormalities. CYP46A1-injected mice had a better performance in the rotarod test and in the footprint patterns measures compared with the GFP-injected control mice. Neuropathologically, there was a reduction in the number of mutant ataxin-3 aggregates and an increase in the number of Purkinje cells upon CYP46A1 expression mediated by lentiviral vectors [49].

Recently, targeted ULK 1/2 expression as an autophagy inducer was investigated as a possible therapeutic strategy for MJD/SCA3. Using an MJD/SCA3 transgenic mouse model [43], ULK 1/2 was injected using lentiviral vectors in the cerebellum. The results showed a rescue of the motor performance of treated mice at 9 weeks post-injection for the swimming and beamwalk tests. Along with this, a major rescue of motor coordination in the rotarod test and an improvement in the ataxic phenotype were observed at 6 weeks post-injection compared with the control group that was injected with GFP. Neuropathological findings showed an increase in the cerebellar volume and in the number of Purkinje cells. Moreover, aggregates were also reduced in the ULK 1/2 injected animals [50].

## 4. Concluding Remarks

MJD/SCA3 is a rare, inherited, monogenic, neurodegenerative disease which remains without treatment. Therefore, there is an urgent and unmet need for disease-modifying therapies, both pharmacological and based on advanced therapies. In the past few years, much effort has been made to understand the molecular pathogenesis of the disease, identify prognostic disease biomarkers, and develop therapeutic strategies. In this last aspect, unfortunately, most of these strategies were only evaluated in a preclinical setting and were not tested in clinical trials with patients. This happens for many non-exclusive reasons ranging from economic issues to a lack of reliable markers to measure therapy outcomes in patients. In fact, much effort has been made to develop studies that try to identify MJD/SCA3 prognostic biomarkers. For this, single-cell technology and spatial transcriptomics technologies, which are now becoming more popular and generalized, can contribute decisively. Both technologies could also provide important hints regarding understanding the disease pathogenesis and, importantly, identifying new therapeutic targets [49]. 

Without any doubt, one of the most studied targets in the field of neurodegenerative diseases is autophagy. In fact, it is established that autophagy dysfunction and impairment underlie the pathogenesis of neurodegenerative diseases, including MJD/SCA3. Therefore, it is not surprising that several studies targeted the activation of autophagy as therapy, both using drugs or gene-based approaches. In this review, we focused on preclinical studies developed for MJD/SCA3 targeting autophagy. While those studies reported promising results, only one was pursued in a clinical trial, in which trehalose was administered to MJD/SCA3 patients. Therefore, a continuous effort must be made to develop preclinical studies with quality and robustness to translate the results obtained to clinical trials and thus offer some hope for patients and their families.

## Figures and Tables

**Table 1:** List of several known ataxin-3 interactors and related molecular functions.

Interactors	Type	Function	References
Fork-head box O	Transcription factor	Binds to promoter regions and interacts with other factors to modulate gene expression	Mohan, R. et al., 2014 [14]
CBP	Co-activator	Induces a nuclear response by cascade signalling	McCampbell, A. et al., 2000 [15]
P300	Histone acetyltransferase	Enhances and regulates gene expression	Evers, M. et al., 2014 [9]
HDAC3	Histone deacetylases	Deacetylases the promoter region, leading to chromatin compaction	Schulze, S. et al., 2007 [17]
Ncor	Co-repressor	Assists the process of histone deacetylase recruitment	Watson, P. et al., 2012 [18]
RAD32	Nucleotide excision repair protein	Inhibits the formation of multi-ubiquitin chain formation and the degradation of proteolytic substrate	Madura, k. et al., 2000 [19]

**Table 2:** Overview of selected studies using pharmacological compounds to activate autophagy as a therapeutic strategy for MJD/SCA3.

Drug	Dose	Delivery System	Model	Reference
Pharmacological				
Rapamycin	0.2 µg/mL	-	African green monkey kidney cells (COS-7)	Ravikumar B. et al., 2002 [37]
	0.2 µg/mL		Rat pheochromocytoma cells (PC12)	
Temsirolimus	20 mg/Kg	Intraperitoneal	Animal model (Torashima et al., 2008 [39])	Menzies F. et al., 2010 [40]
	200 µM	-	(Human neuronal culture) iPSC	
Cordycepin	20 µM	-	Neuroblastoma cells (N2A)	Marcelo A. et al., 2018 [41]
	20 mg/Kg of cordycepinNaCl 0.1%	Intraperitoneal	Animal model (Alves S. et al., 2008 [42])	
	20 mg/Kg of cordycepinNaCl 0.1%	Intraperitoneal	Animal model (Torashima et al., 2008 [39])	
Trehalose	2%	Oral administration	Animal model (Torashima et al., 2008 [39])	Santana M. et al., 2020 [43]
	1 mM, 10 mM, 100 mM	-	Neuroblastoma cells (N2A)	
Carbamazepine	50 mg/Kg	Intraperitoneal	Animal model (Alves S. et al., 2008 [42])	
	100 µM	-	Neuroblastoma cells (N2A)	Vasconcelos Ferreira et al., 2022 [44]
n-Butylidenephtalide	50 mg/kg	Oral administration	Animal model (Cemal et al., 2002 [45])	Lee J. et al., 2021 [46]

**Table 3:** Overview of the main outcomes of the selected studies presented in Table 2 using pharmacological compounds to activate autophagy.

Treatment	Model	Behavior Improvement	Neuronal Loss/Cell Death	Mutant Ataxin-3	Reference
Pharmacological					
Rapamycin	Cell model	NE	Cell death decrease	Less cells with aggregates	Ravikumar B. et al., 2002 [37]
Temsirolimus	Mouse model	Rotarod improvement	NA	Reduced number of aggregatesReduced levels of mutant ataxin-3	Menzies F. et al., 2010 [40]
Cordycepin	Cell model	NE	NE	Reduced levels of mutant ataxin-3	Marcelo A. et al., 2018 [41]
	Mouse model	Rotarod improvement	Preservation of neurons	Reduced number of aggregates	
		Footprint improvement	Increase in the number of Purkinje cells	Decrease in the aggregates’ size	
Trehalose	Cell model	NE	NE	Decreased expression of mutant ataxin-3	Santana M. et al., 2020 [43]
	Mouse model	Rescue of motor coordination	Preservation of Purkinje cells	Decrease in the aggregates’ size	
		Decreased ataxia phenotype		Decrease in the number of aggregates	
Carbamazepine	Cell model	NE	NE	Reduced high levels of the molecular weight species of ataxin-3	Vasconcelos Ferreira et al., 2022 [44]
	Mouse model	Rescue of motor coordination and balance	Purkinje cells preservation	Reduced number of aggregates	
			Molecular layer thickness preservation		
n-Butylidenephthalide	Cell model	NE	NE	Reduced levels of mutant ataxin-3	Lee J. et al., 2021 [46]
	Mouse model	Rotarod improvement	Increase in brain size	Decrease in the number of aggregates	
		Footprint improvement	Preservation of Purkinje cells		
			Preservation of the neurite network		

**Table 4:** Overview of several gene-based approaches targeting the activation of autophagy in MJD/SCA3 models.

Treatment	Dose	Delivery Method	Model	Reference
Gene				
	NA	-	Patients’ fibroblasts	
*BECN1*	200 µg of p24/mL	Intraperitoneal	Animal model (Goti D. et al., 2004)	Nascimento-Ferreira I. et al., 2011 [31]
	10 ng p24 antigen/10^5^ cells	-	Striatal primary cells	
	10 ng of p24antigen/10^5^ cells	-	Neuroblastoma cells (N2a)	
	6 µL of 200,000 ng of p24/mL	Intraperitoneal	Animal model (Torashima et al., 2008 [39])	
*BECN1*	10 ng of lentivirus/100,000 cells	-	Primary cerebellar cells	Nascimento-Ferreira I. et al., 2013 [47]
	10 ng of p24 antigen/10^5^ cells	-	Neuroblastoma cells (N2a)	
	1 × 10^9^ vg/µL of AAVrh10	Intraperitoneal	Animal model (Alves S. et al., 2008 [42])	Nóbrega C. et al., 2019 [48]
*CYP46A1*	NA	-	Neuroblastoma cells (N2a)	
	2 × 10^9^ viral genomes of AAV1/2	Intraperitoneal	Animal model (Torashima et al., 2008 [39])	
*ULK*	60 ng of p24 antigen/1 × 10^5^ cells	-	Animal model (Alves S. et al., 2008 [42])	Vasconcelos-Ferreira et al. 2022 [49]
	60 ng of p24 antigen/1 × 10^5^ cells	-	Human ULK1 KO and control HAP1 cell lines	

NA, not available.

**Table 5:** Overview of the main outcomes of the gene-based strategies used to induce autophagy in MJD/SCA3 mouse models.

Treatment	Model	Behavior Improvement	Neuronal Loss/Cell Death	MutAtaxin-3	Reference
Gene based					
*BECN1*	Mouse model	NE	Neuronal dysfunction improvement	Reduction in the number of aggregates	Nascimento-Ferreira I. et al., 2013 [47]
*BECN1*	Mouse model	Rescue of coordination and balance skills	Neuronal preservation Preservation of Purkinje cellsCerebellar atrophy prevention	Reduction in the number of aggregates	Nascimento-Ferreira I. et al., 2013 [47]
*CYP46A1*	Mouse model	Alleviation of motor phenotype deficits	Neuronal preservation Preservation of Purkinje cells	Reduction in the number of aggregates	Nóbrega C. et al., 2019 [48]
*ULK1*	Mouse model	Rescue of motor performance	Neuronal preservation Preservation of Purkinje cells	Reduction in the number of aggregates	Vasconcelos-Ferreira A. et al., 2022 [49]

NE/NA, not existent/not available.

## Data Availability

Not applicable.

## References

[1] Li T., Martins S., Peng Y., Wang P., Hou X., Chen Z., Wang C., Tang Z., Qiu R., Chen C. (2019). Is the High Frequency of Machado-Joseph Disease in China Due to New Mutational Origins?. Front. Genet..

[2] Ruano L., Melo C., Silva M.C., Coutinho P. (2014). The global epidemiology of hereditary ataxia and spastic paraplegia: A systematic review of prevalence studies. Neuroepidemiology.

[3] Matos C.A., de Almeida L.P., Nóbrega C. (2019). Machado-Joseph disease/spinocerebellar ataxia type 3: Lessons from disease pathogenesis and clues into therapy. J. Neurochem..

[4] Matos C.A., de Macedo-Ribeiro S., Carvalho A.L. (2011). Polyglutamine diseases: The special case of ataxin-3 and Machado-Joseph disease. Prog. Neurobiol..

[5] Bettencourt C., Lima M. (2011). Machado-Joseph Disease: From first descriptions to new perspectives. Orphanet J. Rare Dis..

[6] Koeppen A.H. (2018). The Neuropathology of Spinocerebellar Ataxia Type 3/Machado-Joseph Disease. Adv. Exp. Med. Biol..

[7] Paulson H. (2012). Machado-Joseph disease/spinocerebellar ataxia type 3. Handb. Clin. Neurol..

[8] Chow M.K., Mackay J.P., Whisstock J.C., Scanlon M.J., Bottomley S.P. (2004). Structural and functional analysis of the Josephin domain of the polyglutamine protein ataxin-3. Biochem. Biophys. Res. Commun..

[9] Evers M.M., Toonen L.J., van Roon-Mom W.M. (2014). Ataxin-3 protein and RNA toxicity in spinocerebellar ataxia type 3: Current insights and emerging therapeutic strategies. Mol. Neurobiol..

[10] Evert B.O., Araujo J., Vieira-Saecker A.M., de Vos R.A., Harendza S., Klockgether T., Wüllner U. (2006). Ataxin-3 represses transcription via chromatin binding, interaction with histone deacetylase 3, and histone deacetylation. J. Neurosci..

[11] Macedo-Ribeiro S., Cortes L., Maciel P., Carvalho A.L. (2009). Nucleocytoplasmic shuttling activity of ataxin-3. PLoS ONE.

[12] Lieberman A.P., Shakkottai V.G., Albin R.L. (2019). Polyglutamine Repeats in Neurodegenerative Diseases. Annu. Rev. Pathol..

[13] Mao Y., Senic-Matuglia F., Di Fiore P.P., Polo S., Hodsdon M.E., De Camilli P. (2005). Deubiquitinating function of ataxin-3: Insights from the solution structure of the Josephin domain. Proc. Natl. Acad. Sci. USA.

[14] Mohan R.D., Abmayr S.M., Workman J.L. (2014). The expanding role for chromatin and transcription in polyglutamine disease. Curr. Opin. Genet. Dev..

[15] McCampbell A., Taylor J.P., Taye A.A., Robitschek J., Li M., Walcott J., Merry D., Chai Y., Paulson H., Sobue G. (2000). CREB-binding protein sequestration by expanded polyglutamine. Hum. Mol. Genet..

[16] Li F., Macfarlan T., Pittman R.N., Chakravarti D. (2002). Ataxin-3 is a histone-binding protein with two independent transcriptional corepressor activities. J. Biol. Chem..

[17] Schulze S.R., Wallrath L.L. (2007). Gene regulation by chromatin structure: Paradigms established in Drosophila melanogaster. Annu. Rev. Entomol..

[18] Watson P.J., Fairall L., Schwabe J.W. (2012). Nuclear hormone receptor co-repressors: Structure and function. Mol. Cell Endocrinol..

[19] Ortolan T.G., Tongaonkar P., Lambertson D., Chen L., Schauber C., Madura K. (2000). The DNA repair protein rad23 is a negative regulator of multi-ubiquitin chain assembly. Nat. Cell Biol..

[20] Breuer P., Haacke A., Evert B.O., Wüllner U. (2010). Nuclear aggregation of polyglutamine-expanded ataxin-3: Fragments escape the cytoplasmic quality control. J. Biol. Chem..

[21] Nóbrega C., Simões A.T., Duarte-Neves J., Duarte S., Vasconcelos-Ferreira A., Cunha-Santos J., Pereira D., Santana M., Cavadas C., de Almeida L.P. (2018). Molecular Mechanisms and Cellular Pathways Implicated in Machado-Joseph Disease Pathogenesis. Adv. Exp. Med. Biol..

[22] Nobrega C., Carmo-Silva S., Albuquerque D., Vasconcelos-Ferreira A., Vijayakumar U.G., Mendonça L., Hirai H., Pereira de Almeida L. (2015). Re-establishing ataxin-2 downregulates translation of mutant ataxin-3 and alleviates Machado-Joseph disease. Brain.

[23] Levine B., Kroemer G. (2019). Biological Functions of Autophagy Genes: A Disease Perspective. Cell.

[24] Mizushima N. (2007). Autophagy: Process and function. Genes. Dev..

[25] Glick D., Barth S., Macleod K.F. (2010). Autophagy: Cellular and molecular mechanisms. J. Pathol..

[26] Chandran A., Rochet J.C. (2022). Shining a light on autophagy in neurodegenerative diseases. J. Biol. Chem..

[27] Cortes C.J., La Spada A.R. (2015). Autophagy in polyglutamine disease: Imposing order on disorder or contributing to the chaos?. Mol. Cell Neurosci..

[28] Marcelo A., Afonso I.T., Afonso-Reis R., Brito D.V., Costa R.G., Rosa A., Alves-Cruzeiro J., Ferreira B., Henriques C., Nobre R.J. (2021). Autophagy in Spinocerebellar ataxia type 2, a dysregulated pathway, and a target for therapy. Cell. Death Dis..

[29] Kang R., Zeh H.J., Lotze M.T., Tang D. (2011). The Beclin 1 network regulates autophagy and apoptosis. Cell. Death Differ..

[30] Ashkenazi A., Bento C.F., Ricketts T., Vicinanza M., Siddiqi F., Pavel M., Squitieri F., Hardenberg M.C., Imarisio S., Menzies F.M. (2017). Polyglutamine tracts regulate beclin 1-dependent autophagy. Nature.

[31] Nascimento-Ferreira I., Santos-Ferreira T., Sousa-Ferreira L., Auregan G., Onofre I., Alves S., Dufour N., Colomer Gould V.F., Koeppen A., Déglon N. (2011). Overexpression of the autophagic beclin-1 protein clears mutant ataxin-3 and alleviates Machado-Joseph disease. Brain.

[32] Onofre I., Mendonça N., Lopes S., Nobre R., de Melo J.B., Carreira I.M., Januário C., Gonçalves A.F., de Almeida L.P. (2016). Fibroblasts of Machado Joseph Disease patients reveal autophagy impairment. Sci. Rep..

[33] Komatsu M., Waguri S., Koike M., Sou Y.S., Ueno T., Hara T., Mizushima N., Iwata J.I., Ezaki J., Murata S. (2007). Homeostatic levels of p62 control cytoplasmic inclusion body formation in autophagy-deficient mice. Cell.

[34] Tanida I., Ueno T., Kominami E. (2008). LC3 and Autophagy. Methods Mol. Biol..

[35] Aparicio R., Rana A., Walker D.W. (2019). Upregulation of the Autophagy Adaptor p62/SQSTM1 Prolongs Health and Lifespan in Middle-Aged Drosophila. Cell. Rep..

[36] Klionsky D.J., Abdelmohsen K., Abe A., Abedin M.J., Abeliovich H., Arozena A.A., Adachi H., Adams C.M., Adams P.D., Adeli K. (2016). Guidelines for the use and interpretation of assays for monitoring autophagy (3rd edition). Autophagy.

[37] Ravikumar B., Duden R., Rubinsztein D.C. (2002). Aggregate-prone proteins with polyglutamine and polyalanine expansions are degraded by autophagy. Hum. Mol. Genet..

[38] Bichelmeier U., Schmidt T., Hübener J., Boy J., Rüttiger L., Häbig K., Poths S., Bonin M., Knipper M., Schmidt W.J. (2007). Nuclear localization of ataxin-3 is required for the manifestation of symptoms in SCA3: In vivo evidence. J. Neurosci..

[39] Torashima T., Koyama C., Iizuka A., Mitsumura K., Takayama K., Yanagi S., Oue M., Yamaguchi H., Hirai H. (2008). Lentivector-mediated rescue from cerebellar ataxia in a mouse model of spinocerebellar ataxia. EMBO Rep..

[40] Menzies F.M., Huebener J., Renna M., Bonin M., Riess O., Rubinsztein D.C. (2010). Autophagy induction reduces mutant ataxin-3 levels and toxicity in a mouse model of spinocerebellar ataxia type 3. Brain.

[41] Marcelo A., Brito F., Carmo-Silva S., Matos C.A., Alves-Cruzeiro J., Vasconcelos-Ferreira A., Koppenol R., Mendonça L., de Almeida L.P., Nóbrega C. (2019). Cordycepin activates autophagy through AMPK phosphorylation to reduce abnormalities in Machado-Joseph disease models. Hum. Mol. Genet..

[42] Alves S., Régulier E., Nascimento-Ferreira I., Hassig R., Dufour N., Koeppen A., Carvalho A.L., Simoes S., de Lima M.C.P., Brouillet E. (2008). Striatal and nigral pathology in a lentiviral rat model of Machado-Joseph disease. Hum. Mol. Genet..

[43] Santana M.M., Paixão S., Cunha-Santos J., Silva T.P., Trevino-Garcia A., Gaspar L.S., Nóbrega C., Nobre R.J., Cavadas C., Greif H. (2020). Trehalose alleviates the phenotype of Machado-Joseph disease mouse models. J. Transl. Med..

[44] Vasconcelos-Ferreira A., Carmo-Silva S., Codêsso J.M., Silva P., Martinez A.R.M., França M.C., Nóbrega C., Pereira de Almeida L. (2022). The autophagy-enhancing drug carbamazepine improves neuropathology and motor impairment in mouse models of Machado-Joseph disease. Neuropathol. Appl. Neurobiol..

[45] Cemal C.K., Carroll C.J., Lawrence L., Lowrie M.B., Ruddle P., Al-Mahdawi S., King R.H., Pook M.A., Huxley C., Chamberlain S. (2002). YAC transgenic mice carrying pathological alleles of the MJD1 locus exhibit a mild and slowly progressive cerebellar deficit. Hum. Mol. Genet..

[46] Lee J.H., Lin S.Y., Liu J.W., Lin S.Z., Harn H.J., Chiou T.W. (2021). n-Butylidenephthalide Modulates Autophagy to Ameliorate Neuropathological Progress of Spinocerebellar Ataxia Type 3 through mTOR Pathway. Int. J. Mol. Sci..

[47] Nascimento-Ferreira I., Nobrega C., Vasconcelos-Ferreira A., Onofre I., Albuquerque D., Aveleira C., Hirai H., Deglon N., Pereira de Almeida L. (2013). Beclin 1 mitigates motor and neuropathological deficits in genetic mouse models of Machado-Joseph disease. Brain.

[48] Nóbrega C., Mendonça L., Marcelo A., Lamazière A., Tomé S., Despres G., Matos C.A., Mechmet F., Langui D., den Dunnen W. (2019). Restoring brain cholesterol turnover improves autophagy and has therapeutic potential in mouse models of spinocerebellar ataxia. Acta Neuropathol..

[49] Vasconcelos-Ferreira A., Martins I.M., Lobo D., Pereira D., Lopes M.M., Faro R., Lopes S.M., Verbeek D., Schmidt T., Nóbrega C. (2022). ULK overexpression mitigates motor deficits and neuropathology in mouse models of Machado-Joseph disease. Mol. Ther..

[50] Zhang L., Chen D., Song D., Liu X., Zhang Y., Xu X., Wang X. (2022). Clinical and translational values of spatial transcriptomics. Signal. Transduct. Target. Ther..

